# Emergency admissions of cancer as a marker of diagnostic delay

**DOI:** 10.1038/bjc.2012.423

**Published:** 2012-10-09

**Authors:** W Hamilton

**Affiliations:** 1 Primary care diagnostics, Discovery unit, University of Exeter, Veysey Building, Salmonpool Lane, Exeter EX2 4SG, UK

Almost all commentators accept that the United Kingdom poor performance in cancer is genuine. (Coleman *et al*, 2011) Of more interest is why this should be so; in particular, how much of the problem rests in diagnostic delay? The evidence for diagnostic delay includes poor 1-year mortality (Morris *et al*, 2011), a high number of general practice consultations before diagnosis (Lyratzopoulos *et al*, 2012), and the inverse relationship between the strength of primary care and cancer survival (Vedsted and Olesen, 2011). It is also important to unpick the components of diagnostic delay, as each has different solutions. Delay in seeking medical care can be combated by awareness campaigns; delays in primary care could be tackled by education or enhanced provision of investigations; hospital delays are generally countered by improved organisation of services. Many of these have been tried, with some progress (Health, 2011).

Two papers in this issue examine another marker of possible diagnostic delay – emergency admissions. The first (reference LE-B) deftly merges data from several sources to identify the proportion of cancer patients diagnosed as an emergency. The second (reference AB) examines patient and general practice factors to explain differences in these emergency admission proportions. Both start from the position that emergency admissions – with their higher mortality – may be a failure of the diagnostic process, and may be preventable. If so, then emergency admission proportions could be used as a proxy for quality of the cancer diagnostic process. Better still, emergency admission data are collected routinely, and the algorithms used in Ellis-Brooks *et al* (2012) can be repeated, so could give us a reasonably fast moving indicator of any progress in cancer diagnosis.

Interpretation of emergency admission proportions is not simple. Emergency admissions are defined with respect to time, and not necessarily by severity of the patient’s overall condition, though these may overlap. There is a considerable difference between leukaemia being discovered on a routine blood count, and a patient with bowel obstruction complicating colon cancer. Both patients will generally be admitted to hospital immediately – and be classified as emergencies. Most patients with leukaemia have pallor, fatigue, with perhaps easy bruising, and as long as a blood count is performed reasonably swiftly, no diagnostic improvement is possible. Conversely, the second patient has more than just cancer – he has a complication of the cancer. This may have been a diagnostic failure, in that most patients with such emergencies have described a probable symptom of their cancer to their GP before diagnosis (Cleary *et al*, 2007). However, it may be the first presentation of the illness, or the patient may already have been referred for investigation when the crisis arose. These three possibilities are all amenable to improvement – but the method of improvement will be different for each one. Even so, colorectal emergencies are probably a reasonable proxy for diagnostic underperformance, albeit a crude one. This is not the case at a practice level, however, because the small number of cancer diagnoses in any one practice in a year makes sensible interpretation of cancer diagnostic metrics – including the emergency admission proportion – almost impossible. In any case, the main associations between emergencies and practice characteristics seen in Bottle *et al* (2012) – patient age, ethnicity, and deprivation – are hardly amenable to change.

It is attractive to rank cancers in order of diagnostic underperformance, allowing particular cancers to be prioritised for intervention: both papers make much of the differences between cancers. This may again be the nature of the beast. There is an marked disparity between breast cancer, with emergency admission proportions in the two papers of 4 and 5%, and cerebral tumours at 49 and 62% (as a side issue, these different figures show how important different definitions and methodologies are). Both cancers are dominated by a main symptom – breast lump and headache. The risk of a cancer with a breast lump is high, perhaps 8% (Barton *et al*, 1999), so GPs are encourage to refer all women with breast problems, to ensure all those with cancer are identified swiftly. The very low emergency admission proportion suggests this policy works – at considerable cost, of course (Hamilton, 2008). In contrast, the risk of a brain tumour with a new onset headache presented to general practice is ∼0.09% (Hamilton and Kernick, 2007) so a policy of investigating everyone with new headache would not only overwhelm services, CT scanning could generate more cancers than the lives saved (Hamilton and Roobottom, 2012). GPs have to identify which patients with headache have additional symptoms, and select them for scanning, which is now available directly for GPs (Department of Health, 2012). This is an inexact science: is it any surprise that many patients are diagnosed after a seizure, and thus as an emergency? However, like colorectal cancer, emergency admissions may be a reasonable proxy for speed of diagnosis of brain tumours (and if they fall once the open access MRI scheme matures, this will be welcomed) but they will surely never remotely approach the proportion seen in breast cancer.

Mortality is higher in patients presenting with emergencies. Again, we must be cautious in attributing all the higher mortality to the emergency itself. Some may be ‘reverse causation,’ whereby patients with advanced cancer at first presentation are rapidly identified and hospitalised. These so-called ‘sick-quick’ had a high inherent mortality anyway. This may partly explain the waiting time paradox, where short times to diagnosis have a higher mortality (Torring *et al*, 2011). This does not mean that emergency admissions should be ignored – more that the potential mortality benefit from a reduction may be less than hoped.

Overall, some emergency admissions are preventable by earlier diagnosis. Thus, the slow fall in the proportion of emergency admissions in the United Kingdom is good news (National Cancer Intelligence Network, 2012). However, the measure is a crude proxy for quality of the diagnostic process, and certainly too crude to be used at a local level. It is also of very limited value in comparisons across cancer sites. Its greatest value will be in monitoring the effect of initiatives targeting a single cancer site over time.
